# Contextual imitation of intransitive body actions in a Beluga whale (*Delphinapterus leucas*): A “do as other does” study

**DOI:** 10.1371/journal.pone.0178906

**Published:** 2017-06-21

**Authors:** José Z. Abramson, Mª Victoria Hernández-Lloreda, José-Antonio Esteban, Fernando Colmenares, Francisco Aboitiz, Josep Call

**Affiliations:** 1 Departamento de Psiquiatría, Facultad de Medicina, y Centro Interdisciplinario de Neurociencia, Pontificia Universidad Católica de Chile, Santiago, Chile; 2 Centro de Estudios Avanzados, Universidad de Playa Ancha, Valparaíso, Chile; 3 Grupo UCM de Estudio del Comportamiento Animal y Humano Universidad Complutense de Madrid, Madrid, Spain; 4 Departamento de Metodología de las Ciencias del Comportamiento, Facultad de Psicología, Campus de Somosaguas, Universidad Complutense de Madrid, Madrid, Spain; 5 Research Department, Parques Reunidos Valencia S. A. Oceanogràfic, Ciudad de las Artes y las Ciencias, Valencia, Spain; 6 Departamento de Psicobiología, Facultad de Psicología, Campus de Somosaguas, Universidad Complutense de Madrid, Madrid, Spain; 7 School of Psychology and Neuroscience, University of St Andrews, St Mary’s Quad, South Street, St Andrews, Fife KY, United Kingdom; 8 Department of Developmental and Comparative Psychology, Max-Planck Institute for Evolutionary Anthropology, Deutscher Platz 6, Leipzig, Germany; University of Portsmouth, UNITED KINGDOM

## Abstract

Cetaceans are remarkable for exhibiting group-specific behavioral traditions or cultures in several behavioral domains (e.g., calls, behavioral tactics), and the question of whether they can be acquired socially, for example through imitative processes, remains open. Here we used a “Do as other does” paradigm to experimentally study the ability of a beluga to imitate familiar intransitive (body-oriented) actions demonstrated by a conspecific. The participant was first trained to copy three familiar behaviors on command (training phase) and then was tested for her ability to generalize the learned “Do as the other does” command to a different set of three familiar behaviors (testing phase). We found that the beluga (1) was capable of learning the copy command signal “Do what-the-other-does”; (2) exhibited high matching accuracy for trained behaviors (mean = 84% of correct performance) after making the first successful copy on command; (3) copied successfully the new set of three familiar generalization behaviors that were untrained to the copy command (range of first copy = 12 to 35 trials); and (4) deployed a high level of matching accuracy (mean = 83%) after making the first copy of an untrained behavior on command. This is the first evidence of contextual imitation of intransitive (body-oriented) movements in the beluga and adds to the reported findings on production imitation of sounds in this species and production imitation of sounds and motor actions in several cetaceans, especially dolphins and killer whales. Collectively these findings highlight the notion that cetaceans have a natural propensity at skillfully and proficiently matching the sounds and body movements demonstrated by conspecifics, a fitness-enhancing propensity in the context of cooperative hunting and anti-predatory defense tactics, and of alliance formation strategies that have been documented in these species’ natural habitats. Future work should determine if the beluga can also imitate novel motor actions.

## Introduction

Cetaceans are long-lived, large-brained, cognitively advanced, and highly sociable, and flexibly cooperative animals [1–3] that live in ecological scenarios where the problems of survival (as predators and preys) and reproduction are better solved socially [4,5]. One of the characteristic features that make cetaceans all the more remarkable is their group-specific behavioral signatures [4–7] including vocal repertoires and hunting and foraging tactics that do not seem to be either ecologically or genetically inherited.

Classification schemes of social learning abound and coincide only partially [8–13]. One broad definition states that social learning is acquiring knowledge about the animate and inanimate world that is influenced by observation of, or interaction with, another individual or its products. However researchers generally agree that social learning is not a unitary process, and the published taxonomies of social learning explicitly acknowledge that different forms of social learning can potentially be driven by psychological processes that vary in its computational demands [8–16].

Imitation is generally believed to be a cognitively demanding form of social learning, which can be broken down into several categories that differ in the complexity of their cognitive underpinnings [9,11,13,15, 17–22]. Broadly defined, an individual (i.e., the subject or observer) can be said to imitate when it matches the demonstrated behavior of another individual (i.e., the demonstrator). However, the demonstrated behavior that is copied by the observer can be *familiar* versus *novel*, and *transitive* (object-oriented) versus *intransitive* (body-oriented). These distinctions may well reflect the engagement of different cognitive processes [17–24]. For example, performing an action that is already present in the subject’s repertoire in response to seeing it done by a demonstrator is thought to be cognitively less demanding than if the demonstrated action is entirely novel. Thus, some researchers have argued that the former, called *contextual learning* [25] can be accounted for by relatively simple cognitive processes such as response facilitation or emulation, and that the latter, called *production learning* [25], is the only form of true imitation that is founded on higher-level cognitive skills [19,23]. Similarly, the copying of so-called transparent or transitive actions is hypothesized to engage cognitive skills that can (at least partly) be different from those required to match opaque or intransitive actions [11,15,22,24,26]. Perhaps this may explain the mixed results obtained in experiments on contextual imitation in dogs that appeared to be influenced by whether the demonstrated actions were transitive [27] or intransitive [28,29].

Most experimental studies on cetacean social learning, and especially imitation, have focused mainly on the bottlenose dolphin (*Tursiops truncatus*) [30–33]. Collectively, the main conclusion from these studies is that dolphins are skillful imitative generalists quite capable of copying vocal and motor behaviors demonstrated by conspecifics, by humans and even generated by computers. This evidence suggests that bottlenose dolphins are one of the few non-human animal species capable of the capacity for multimodal (vocal and action) imitation. It has been suggested that the detachment from modality-specific inputs may represent a substantial change in neural organization, one that affects not only imitation but also communication [34].

Recently, production imitation of (novel) motor behaviors has also been reported in an experimental study of another delphinid species, the killer whale, *Orcinus orca* [35]. The present experimental study of (familiar) motor imitation focuses on another toothed cetacean, the beluga *(Delphinapterus leucas)*, a species whose social behavior is poorly known [4,5]. Two recent studies have reported that belugas can imitate sounds from a variety of sources, including human speech [36,37]. Ridgway et al. [36] reported that a beluga spontaneously imitated human sounds and investigated the physical mechanisms that the beluga used to produce speech-like sounds. Murayama et al. [37] tested the ability of a male beluga to copy familiar conspecific sounds, novel artificial (computer-generated) sounds and human speech. They found that their study subject succeeded at imitating both familiar and novel sounds.

In the present preliminary study we used a Do-as-the other-does paradigm to test the ability of a female beluga to exhibit contextual learning of familiar, (but untrained to a copy command), intransitive body-oriented motor actions. The Do as I do method, originally used by Hayes and Hayes (1952) [38] in a study of motor imitation in a home-raised chimpanzee, involves copying another’s action under a specific signal (‘Do this!’), without any other scaffolding information (e.g., results-based cues). Some authors have argued that to solve this task the animal subjects need to have some kind of concept of imitation, as the method depends on the generalization of a trained signal to a conceptual order that is ‘copy what I am (or what the other) is doing’ [9,39]. In fact no success has been achieved in trying to train a macaque monkey to imitate on request [40]. However this training technique has been successfully used in several species of great apes [41–44], dogs [45,46], and cetaceans (killer whale [35], dolphins [47–50], and a beluga [37].

## Material and methods

### Subjects

The participants in this study were two beluga whales (*Delphinapterus leucas*) housed at L’Oceanografic Aquarium in Valencia, Spain: an 18 year old female named Yulka and a 55 year old male named Kairo. Yulka was the experimental subject or observer and Kairo served as the demonstrator. Yulka was mother-reared and captured when she was 1-year-old in the Okhotsk Sea. She had been housed with Kairo in the same pool since 2003. Each subject was daily fed approximately 18 kg of freshly thawed herring, hake, capelin, and pota, one half of which was typically consumed during experimental sessions. Yulka had already been trained to produce 53 examination and exercise behaviors using standard operant conditioning procedures. Prior to this experiment, both subjects participated in biological studies and veterinary procedures, but only Yulka had participated in cognitive studies (Abramson et al.[35]). Subjects were never food deprived in any way, regardless of performance.

### Ethics statement

The Ethics and Animal Welfare Committee (CEBA-MEDUC) of the School of Medicine, Pontifical Catholic University of Chile, have approved this research. This research adhered to the legal requirements of the country (Spain) in which the work was carried out and all institutional guidelines.

### Procedure

#### General procedure

Experimental sessions consisted of 6–12 trials, lasting approximately 10–20 min altogether. Some sessions finished earlier if the participants were distracted or disinclined to participate (this only occurred in two sessions). To run the experiments two trainers were needed, one to give the trained signal to the demonstrator (T_D_) and another to give the copy command to the subject (T_S_). The experiment was conducted in the same pool (≈3582 m^3^ and ≈ 800 m^2^) as in a previous cognitive study (Abramson et al.[35]). The pool was equipped with a floating pontoon that measured 2 × 3 m on which the trainers stayed and to which the subjects could be called. The pontoon was attached to the pool wall. The subjects were positively rewarded with fish or pota and with a whistle signal (bridge) whenever they produced a correct response. They received no positive reinforcement following errors, though, and the trials were repeated once again before moving to the next behavior, with the constraint that no more than three test trials of the same familiar action could occur in a row. Reinforcement of the demonstrator beluga was not contingent upon the response of the subject.

The study comprised two phases. 1) Training phase: involved training the subject to respond to a visible gesture-based command “copy” (“Do what the demonstrator is doing”) given by T_S_. This generic "copy" hand sign gesture was made up for this purpose and was the same over all presentations. The signal consisted of T_S_ wielding the right hand and touching with it the open palm of the left hand (see supplementary information). 2) Testing phase: involved testing the subject’s generalization of the T_S_’s copy command to other behaviors performed by the demonstrator. All the behaviors themselves were already part of both individuals’ behavioral repertoire, that is, the subject’s and the demonstrator’s, but they had never been trained (associated) with the copy command.

Therefore, in this experiment we selected behaviors that were already known to the subject and the demonstrator. They were body-oriented actions, that is, intransitive actions. This limited our study to six behaviors in total. These six behaviors performed by the demonstrator were grouped into two categories: a) the three *trained behaviors* used when the subject was being trained to respond to the copy command given by T_S_ (Training phase) and, subsequently interspersed during the testing phase, when the subject had already learned to respond to T_S_’s copy command and was requested to do what the demonstrator was doing and b) the three *untrained behaviors* that were used during the testing phase, but not during the training phase. Table 1 gives the complete list of behaviors examined in this study, and Table 2 gives a summary of the total number of trials for each behavior tested in each phase. All behaviors used in this study were not part of the beluga whales’ natural repertoire but the result of the set of training and controlled by gesture-based commands. All sessions were videotaped by a video camera located above the tank in a position that provided a full view of the two subject–trainer pairs and the entire tank.

**Table 1:** Behaviors tested in each phase.

Training phase 1	Description
Dance (DA)	Rise vertically on water, half of the body on the surface, and roll continuously in 360°
Greeting Tail (GT)	Dive downward to a vertical position with tail fluke protruding from the water and shaking it
Squirt (SQ)	Split water out of the surface
**Testing phase 2**	
Fast Swimming (FS)	Swim in fast mode doing a full 360° circle around the pool
Tail Splash (TS)	Slap tail continuously on water surface
Roll Over (RO)	Turn over, ventral side up, horizontally (parallel to the water surface), and maintain the position

Every behavior is described taking as the starting point the animal facing the trainer while lying horizontally on the water’s surface and in perpendicular position to the pool wall.

**Table 2:** Total number of trials.

	No. of trials only copy signal	First trial copied (only copy signal)	No. of correct trials after 1st copy	% correct after 1st copy
**Phase 1 Training**				
DA	37	19	15/18	83%
GT	36	21	13/15	86%
SQ	40	23	14/17	82%
	No. of trials	First trial copied	No. of correct trials after 1st copy	% correct after 1st copy
**Phase 2 Testing (generalization)**				
FS	74	12	62/62	100%
TS	73	35	25/38	66%
RO	70	17	45/53	85%

Total number of trials for each behavior tested in each phase, number of trials until the demonstrator’s behavior was copied by the subject, number of correct trials after first copy and percent of correct trials after first copy. For familiar trained behaviors we are including data from the 5th session, for trials when the panel was introduced and only the copy signal alone was gradually given.

#### Training phase

**Fig 1:**
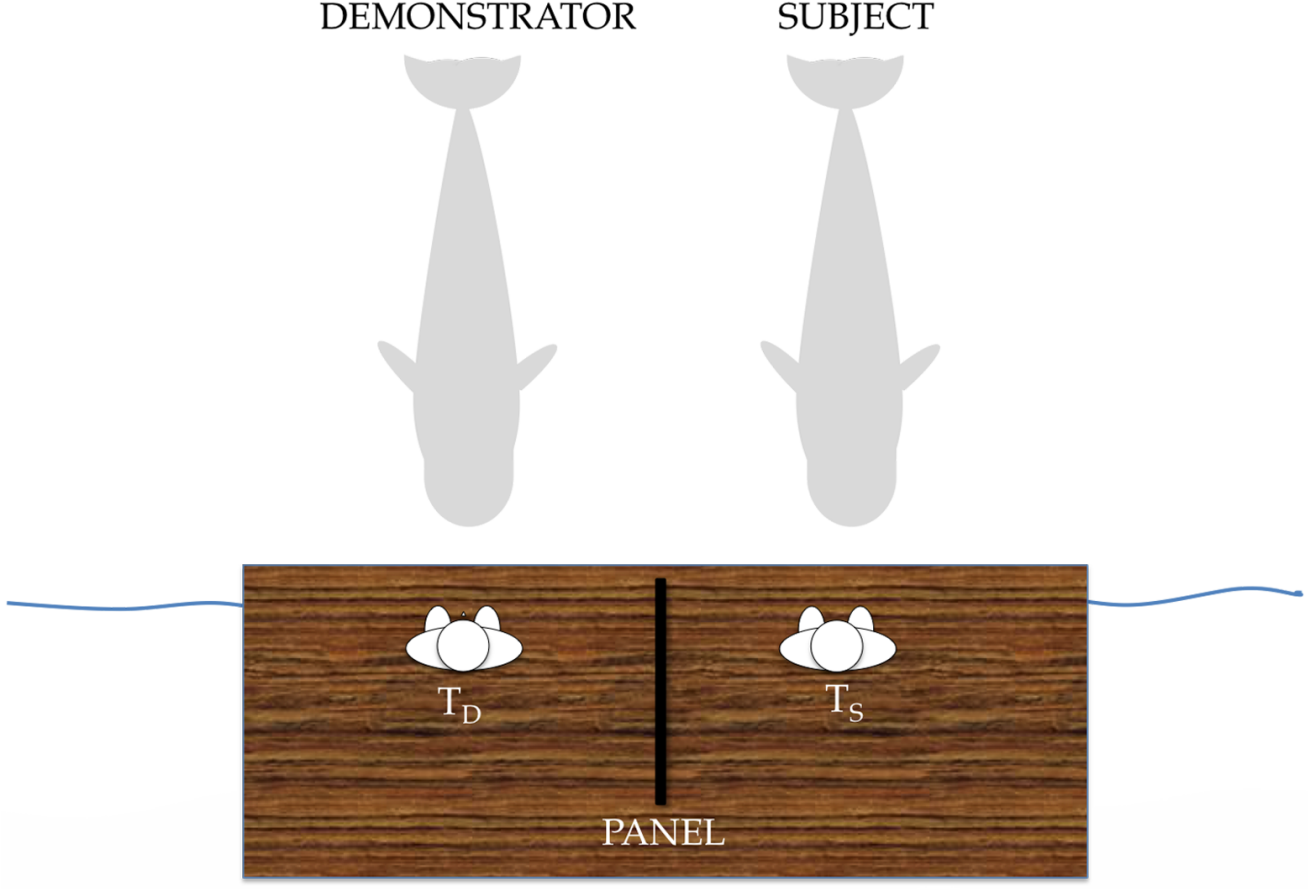
Experimental set up. Two trainers (T_D_ and T_S;_ D for demonstrator and S for subject), were positioned on different sides of an opaque panel 2m long x 91cm high placed in a position in which S and D could see each other and their own trainer, but could not see the other trainer’s commands.

The aim of this first phase was to teach the subject the copy command (“Do as the other does”) given by her trainer (T_S_). The two individuals, the demonstrator and the subject, were positioned next to each other, side by side, in the same pool where the two trainers (T_D_ and T_S_), also placed side by side, without the panel, were facing the demonstrator and the subject, respectively (See Fig 1). Sessions consisted of 1 to 4 control (non-copy) trials and 6–12 training “copy signal” trials. Three familiar behaviors were selected to train the copy signal: 1) DA, 2) GT and SQ. (See Table 1 and S1 and S3 Videos). The criteria required for learning the copy command in this first phase was that the subject reached a correct performance using just the signal in more than 80% of trials.

We began the copy command training with two behaviors, DA and GT. In the first training session, the two individuals were positioned side by side, which meant that the subject could see the signal the T_D_ gave to the demonstrator, and was allowed to perform these two behaviors in tandem with the demonstrator. In the second training session, the demonstrator was first required to perform a selected familiar behavior by T_D_ but T_S_’s signal (the same as for the demonstrator) was delayed for 2–4 seconds relative to the completion of T_D_'s signal. In the third and fourth training sessions, T_S_ inserted the gestural copy command prior to the behavior-specific signal on imitation trials (copy signal + behavior-specific signal). In session five, we also used another familiar behavior, SQ and an opaque panel of 2 m x 1.5 m was erected between the two trainers (See Fig 1). This panel prevented the subject from seeing what T_D_ was signaling to the demonstrator. Several pretests were previously done with the trainers positioned in the water in the same position as the animals to ensure that the subject could only see the behavior of the demonstrator and her own trainer’s signals, but not the signals of the demonstrator’s trainer. This panel also prevented each trainer from seeing what the other was signaling to the other whale. T_D_ was positioned on the left side of the panel, and T_S_ was on the right side. The chief trainer and one of the researchers judged the correctness of each trial and told T_D_ and T_S_ to reinforce or not the subject.

Before introducing the panel, the subject received 54 training trials with the copy signal plus the respective behavior signal (24 trials DA, 24 trials GT and 6 trials SQ). From the fifth session onward, the signals associated with specific behaviors were gradually removed until the 10th session when only the copy signal was used. From then onwards, two more sessions were run using just the copy signal for these three trained behaviors that were randomly presented. The criterion required to reach a correct performance, (80% across two consecutive sessions), was reached in the 11^th^ and 12th session, (that is after having performed, from the 5th to the 10^th^ session 65 trials with the copy signal alone). Two additional sessions were done resulting in Yulka receiving 14 training sessions with a total of 113 training trials with the copy signal alone (37 for DA, 36 for GT and 40 for SQ), (see Table 2 for details).

#### Testing phase (generalization)

The aim of this second phase was to test whether Yulka was able to generalize the “copy” signal to other familiar but untrained behaviors belonging to the subjects’ repertoire of actions performed on command: 1) FS; 2) TS and 3) RO (see Table 1). It is important to highlight that although they were familiar, however, the behaviors used in this training phase had never been associated with the copy or "Do that" signal; in that sense, and unlike those used in phase 1, this new set of behaviors were "untrained". These three untrained actions were introduced one at a time across sessions, and once introduced they were distributed both within and across sessions resulting in a total of 217 testing trials altogether (see Table 2 and S4 and S6 Videos for details). During testing sessions, the 3 trained behaviors already used in the training phase were also interspersed and randomly presented. The already mentioned constraint of no more than 3 trials of the same action in a row was maintained. The criteria required for generalizing the copy command (“Do what the other does!”) for these three untrained behaviors was that the subject performed above chance in producing exact matches of the three behaviors (see below for what we considered chance level performance).

The set-up used was the same panel/trainer configuration as that used during the last training sessions. These testing sessions consisted of 1–4 control trials and 6–12 test trials. We introduced two types of control trials. In *type 1 “noncopy” control tria*ls the subject was asked to perform a different action from the one performed by the demonstrator. These were meant to served three purposes: 1) to maintain the subject’s attention to her own trainer, 2) to avoid her moving from the starting position and her looking at the other trainer’s signals, and 3) to maintain the subject’s motivation, as the correct performance of known behaviors yielded rewards. The behaviors were presented with the constraint that no more than three test trials of the same familiar action could occur in a row. In *type 2 “copy” control trials* the subject was requested to not do anything and subsequently to perform one or two trained behaviors while the demonstrator was performing a different test behavior. This was done right before she was commanded to copy the specific untrained behavior that the demonstrator was performing. This control is more demanding than the former one as the subject is made to maintain the attention to her own trainer (T_S_) while the demonstrator is performing the behavior and is signaled to copy the demonstrated action only when the trainer gives the copy command. This control ensures that the subject responds to the copy command and not to the demonstrated action itself (See S7 Video).

## Data coding and analysis

Coding was done by two experimenters. One experimenter coded the sessions in real time while running the experiment, and recorded for each trial whether the subject’s action was a correct or inaccurate match of the demonstrator’s action. For reliability analysis, a second experimenter watched 70% of a randomly chosen set of videos of each test trial several months after the study had been completed and recorded whether the subject’s action was a correct match of the demonstrator’s action. Inter-observer reliability was found to be very high (Cohen’s kappa was 0.96; P < 0.001; Observer agreement = 0.99). Exact binomial tests were used to establish whether the subject successfully matched the demonstrator’s actions above chance. For the analyses we assumed that chance performance would be successful matching on 1/[number of different familiar behavior requested to be performed + (possibility of doing nothing)] trials. Therefore we assumed a chance performance for Yulka equals to 1/5 for FS, the test behavior introduced in the first place; 1/6 for TS, the test behavior introduced next and 1/7 for RO, the last test behavior. For analyzing the actions jointly we used the most conservative criterion (e.g., level of chance equals 1/5). Note that this is a rather strict criterion given that, in theory, the subject had the possibility to perform any other action from the repertoire of 53 familiar behaviors trained by the trainers and requested usually as part of their training exercises, rather than just those requested in the test situation. Šidák adjustments for multiple exact binomial tests performed by Yulka were used to achieve a family-wise alpha of 0.05.

## Results

### Training the “Do it!” command

Considering all training trials the behaviors were first copied on command in trials 43th (DA), 45th (GT) and 29th (SQ). Considering only trials in which the copy signal was given alone (the signal of the other trainer was no longer visible), they were copied in trials 19^th^ (DA), 21^nd^ (GT) and 23^th^ (SQ) (See Table 2). The subject reached the criterion set to go to the test phase in the 12th session (80% in the 11^th^ and 90% in the 12^th^), that is, after having performed 65 trials distributed across 6 sessions (from the 5^th^ to the 10^th^). From the 11^th^ session onwards the percentage of accuracy in the copy was 85% (in a total of 48 trials distributed across 4 sessions). As shown in Table 2, after the first accurate matching when the copy command alone was introduced (i.e., from 5th session onwards), DA was fully copied in 83% of trials (n = 37), GT in 86% of trials (n = 36), and SQ in 82% of trials (n = 40) (see Table 2).

### Generalizing the “Do as the other does” copy command to untrained demonstrated behaviors

For the three behaviors that were not trained with (associated to) the copy signal introduced in the testing phase, the subject performed above chance in the whole study, producing full matches for 64% of demonstrated behaviors (*P* < 0.001). Analyzing each test behavior separately, Yulka performed significantly above chance in all behaviors, FS: 85%, TS: 36% and RO: 66% (exact binomial tests with Šidák adjustments, all *Ps* < 0.001). FS, the first behavior tested, was copied in the 12th trial (see Table 2 and S4 Video). TS, the second behavior tested, was copied in the 35th trial (S5 Video). Finally, RO was copied in the 17^th^ trial (S6 Video). Yulka’s correct performance after first accurate matching was 100% for FS (n = 62); 66% for TS (n = 38) and 85% for RO (n = 53) (see Table 2).

Finally, in control trials (n = 117), performance was 96% correct. In control trials type 2 with control behaviors interpersed (n = 63), Yulka performed remarkably above chance, producing full matches for 85% of demonstrated actions (p < 0.001). Analyzing the actions separately, RO behavior was copied in 87% of trials (13 out of 15); TS in 91% (10 out of 11 trials), and FS in 100% (38 out of 38 trials) (exact binomial tests with Šidák adjustments, all *Ps* < 0.001).

## Discussion

The beluga whale *(Delphinapterus leucas)* participating in the present experiment was capable of learning the “Do as the other does” command for copying familiar actions. Although other “Do as I Do” studies have reported successful motor imitation, the speed at which individuals learned the copy command has been found to vary highly across studies. For example, compared to dolphins Yulka’s achieved success relatively quickly as Bauer and Johnson [47] reported that their two participant dolphins took “hundreds of trials” and “more than 1,000 trials”, respectively, to learn the mimic command. In contrast, compared to killer whales Yulka’s did it slower as Abramson et al.’s [35] reported that three killer whales started copying the demonstrator’s actions from the very beginning. One possible reason for the difficulty experienced by some species to learn this command is that the success in this task relies on the conceptual learning that may underpin the generalization of this trained “copy what I am (or what the other) is doing” signal to different behaviors [9,39]. Nevertheless, after Yulka’s produced the first correct copy, her matching accuracy remained high (Table 2) in a similar way as dolphins in Bauer and Johnson [50] (81% and 84%) and killer whales in Abramson et al. [38] (83%, 81% and 94%).

Once the beluga of this study learned the copy command she was also capable of generalizing it to other familiar actions, succeeding in copying other 3 familiar, but untrained behaviors, performed by the conspecific demonstrator (i.e., 100%). She did so rather quickly, and her level of correct performance remained high after making the first correct matching (Table 2). Unfortunately, systematic comparisons between studies are difficult because studies vary in terms of the criterion of correct performance, the number and type of actions performed by the demonstrator (e.g., vocal versus motor; transitive versus intransitive), and the kind of demonstrator are also variable (e.g., conspecific, human, computer). For example, compared to dolphins Yulka´s level of correct performance remained high after making the first correct matching (Table 2) as in Jaakkola et al.’s [49] study of motor imitation the researchers found that in the sighted condition (similar to our study) the dolphin’s matching accuracy of 19 motor behaviors was 61%. On the other hand, compared to killer whales, Yulkas’s generalization of the copy command was slower as in the Abramson et al.’s [35] study, familiar behaviors performed by the demonstrator were copied before the 8^th^ trial and many of them on the first attempt (range = 57–93%).

Even though the imitation of familiar action has been documented in several species, all the familiar motor actions that were used and successfully copied in our study were mostly perceptually opaque. Furthermore, they were intransitive, that is, they were body-oriented rather than object-oriented [23,24]. It has been argued that what makes imitation of opaque actions a more difficult achievement is the difference between the information that is available to the observer’s senses when the body movement is performed by the demonstrator and when it is performed by the observer [22–24,26]. It seems that with intransitive actions this difference increases, as the observer does not have too many environmental changes that could serve as cues for guiding his perceptual representation of the action to be matched. Results from previous experimental studies in animal imitation done in apes [41,42] and dogs [28, 45,46] suggest that familiar (usually opaque) intransitive actions are harder to imitate than familiar transitive actions.

In the present study, the beluga watched the body movements performed by the demonstrator and, upon receiving the copy command (“Do what the other is doing”), responded by choosing an untrained action to the copy command from her behavioral repertoire that best matched the demonstrated action. She also did so in the more demanding type 2 copy control trials when the subject was requested not to do anything and subsequently was commanded to perform one or two trained behaviors while the demonstrator was performing a different test behavior. In some type 2 copy control trials Yulka was asked to do nothing and then only when the demonstrator completed his action and she was commanded to copy it she then did it. Overall, our results do not quite fit social learning cognitive mechanisms such as “response facilitation” (the presence of a conspecific performing an act already in the observer’s repertoire increases the probability of an animal which sees it doing the same), or “emulation” (the copying of the results or effects on the environment) [19]. We rule out social facilitation because albeit the subject’s behavior indeed was a copy of the behavior performed by the demonstrator and was already known to her, she actually chose it from across a wide repertoire of other potential behavioral options, she did it upon being given the copy command [30]. We also rule out emulation because in our study, the behaviors performed by the demonstrator were intransitive, that is, they were body-oriented rather than object-oriented [23,24]. Another possibility is that the subject would have learned to respond through the formation of behavior-specific associations rather than through generalization of the copy command to the untrained behaviors. This is suggested by the fact that the subject’s initial copy in the testing phase was found to be no faster than when she first copied in the training phase. However, this would only apply to one of the three behaviors, namely, TS, which took the individual 35 trials after copying. In the other two behaviors, in contrast, this was faster. Moreover, recall that the subject had undergone a training procedure in which the copy signal was associated with the signal for the behavior and presented without a panel. Despite that, the number of trials required until the first copy was produced was reduced for two of the behaviors, namely FS and RO, which only took 12 and 17 trials to be copied, respectively, *versus* 43, 45 and 29 for the three behaviors copied in the training phase, DA, GT and SQ, respectively. This thus strengthens the view that the subject successfully “generalized” the copy command to the new behaviors in the training phase. Future studies with “novel” transfer behaviors (production imitation) are necessary to provide stronger evidence about this generalization capacity.

We realize that our study has important limitations, due to the fact that all the data were collected from only two individual whales in captivity. Experimentally studying the cognition of large marine mammals under controlled conditions constitutes a formidable challenge. This partly explains why most experimental of cognition in marine mammals have tended to rely on very few individuals like in the study reported here (e.g. 35–37; 47–50). Nevertheless, this work complements that conducted in the field by contributing to elucidate candidate mechanisms that might explain some of the behavioral variation observed in field populations [4,5].

In sum, a beluga whale was capable of copying a conspecific’s familiar intransitive and opaque body movements (i.e., contextual imitation) on command. As such, this preliminary study contributes to the growing body of information available on motor and vocal imitation in various cetacean species (bottlenose dolphin [47–50], killer whale [35], beluga whale [36,37]). Moreover, it indicates that beluga whales, similar to dolphins, killer whales, dogs and apes, can be trained to imitate trained actions on command in a “do-as-I-do” task. Although beluga whales can imitate novel sounds [36,37], it remains to be established whether they also shares with dolphins and killer whales the ability to imitate novel intransitive and opaque actions (i.e., productive imitation).

## Supporting information

S1 Video Dance copy example. (M4V)

S2 Video Greeting tail copy example. (M4V)

S3 Video Squirt copy example. (M4V)

S4 Video Fast swimming copy example. (M4V)

S5 Video Tail splash copy example. (M4V)

S6 Video Roll over copy example. (M4V)

S7 Video Control type 2 example. (M4V)

S1 Excel Raw underlying data. (XLSX)
